# Chemokines and Chemokine Receptors: Orchestrating Tumor Metastasization

**DOI:** 10.3390/ijms20010096

**Published:** 2018-12-27

**Authors:** Elisabetta Marcuzzi, Roberta Angioni, Barbara Molon, Bianca Calì

**Affiliations:** 1 Department of Biomedical Sciences, University of Padova, 35131 Padova, Italy; elisabetta.marcuzzi@phd.unipd.it (E.M.); roberta.angioni@studenti.unipd.it (R.A.); barbara.molon@unipd.it (B.M.); 2 Fondazione Istituto di Ricerca Pediatrica Città della Speranza, 35127 Padova, Italy; 3 Institut Curie, 75005 Paris, France

**Keywords:** chemokine, cancer, angiogenesis, tumor immunity, metastasis

## Abstract

Metastasis still represents the primary cause of cancer morbidity and mortality worldwide. Chemokine signalling contributes to the overall process of cancer growth and metastasis, and their expression in both primary tumors and metastatic lesions correlate with prognosis. Chemokines promote tumor metastasization by directly supporting cancer cell survival and invasion, angiogenesis, and by indirectly shaping the pre-metastatic niches and antitumor immunity. Here, we will focus on the relevant chemokine/chemokine receptor axes that have been described to drive the metastatic process. We elaborate on their role in the regulation of tumor angiogenesis and immune cell recruitment at both the primary tumor lesions and the pre-metastatic foci. Furthermore, we also discuss the advantages and limits of current pharmacological strategies developed to target chemokine networks for cancer therapy.

## 1. Introduction

Metastasis, the dynamic process of cancer cell dissemination from the primary tumors to distant organs, still represents the primary cause of cancer morbidity and mortality [1].

To metastasize, tumor cells have to leave the primary tumor site, enter the bloodstream or lymphatic system and seed to other tissues in the body. Importantly, metastasis is a non-random process as it allows circulating tumor cells (CTC) to seed at specific distant tissues. Certain organs, such as the liver, lungs, brain, lymph nodes, and bone marrow are in fact preferential sites for metastasis, while others, such as kidneys, pancreas, and skin, are less frequently affected [2]. According to the “seed and soil” theory developed by Paget in 1889, in order to successfully metastasize, cancer cells have to be able to respond to attractive signals that drive them towards the pre-metastatic sites, and, moreover, they should be able to survive and sprout at their destination site [3,4]. This theory, together with all the animal models developed and clinical retrospective studies, has strongly contributed to explain why specific tumor cells preferentially metastasize to lymph nodes or other tissues on the basis of the receptors expressed on their cell surface [3]. Additionally, continuously rising clinical and experimental evidence has revealed a crucial role for stromal and immune cells colonizing the pre-metastatic sites in supporting cancer cell spreading [5].

Chemokines, or chemotactic cytokines, are small chemoattractant secreted molecules regulating cell positioning and cell recruitment into tissues, playing a pivotal role in embryogenesis, tissue development, and immune response. Chemokines and their receptors are in fact essential mediators of directed migration of leukocytes as well as cancer cells [6]. Approximately 50 chemokines and 20 chemokine receptors have been discovered so far, and, besides their well-characterized functions in immune cell migration and inflammation, they also play a critical role in tumor initiation, promotion, and progression [7,8].

It has been largely reported that chemokine signalling is involved in the regulation of tumor cell choice of if and where they metastasize [9]. Indeed, the identification of a peculiar chemokine receptor array on cancer cells supports the concept that chemokines are able to define the secondary destination of disseminating tumor cells.

Chemokines can be widely divided into two major groups based on their prominent functions: inflammatory and homeostatic chemokines. Among inflammatory chemokines, which are induced by inflammation, we can mention CXCL1, CXCL2, CXCL3, CXCL5, CXCL7, CXCL8, CXCL9, CXCL10, CXCL11, and CXCL14. On the other hand, homeostatic chemokines such as CCL14, CCL19, CCL20, CCL21, CCL25, CCL27, CXCL12, and CXCL13, are constitutively expressed and are involved in homeostatic leukocyte trafficking. Chemokine receptors, which are seven transmembrane spanning proteins coupled to G-proteins, are similarly divided into subfamilies based on their cysteine residues pattern: CXC, CC, CX3C, where C stands for cysteine and X represents non- cysteine amino acids [10]. Importantly, there is a significant ligand promiscuity among chemokine receptors, as some chemokines can bind to and signal through several chemokine receptors, both canonical and atypical ones [11]. 

Inappropriate chemokine/chemokine receptor expression and regulation have been linked to many diseases, especially those associated with inflammation, such as cancer, where chemokine receptor expression often correlates with prognosis. [12] In fact, many chemokines, as well as their receptors, have been detected in both primary tumor lesions and metastatic sites [13].

Chemokines can directly promote cancer cell migration and indirectly support metastatic dissemination by shaping the tumor microenvironment. Chemokines promote the proliferation and survival of tumor cells by different mechanisms, including the induction of mitogen-activated protein kinase (MAPK)/extracellular signal-regulated kinase (Erk) signalling pathways, promoting the expression of important growth-stimulating genes, such as cyclins D1 [14], Fos [15], and heparin-binding epidermal growth factor (HB-EGF) [16]. Furthermore, chemokines can increase the expression of anti-apoptotic genes, such as Mdm2 [17], and negatively modulate Bcl-2 expression and caspase-3 and caspase-9 activation [18]. Notably, by promoting angiogenesis and leukocyte recruitment and activation into the pre-neoplastic niches, chemokines contribute to tumor immunity both in the primary lesions and, more importantly, at the metastatic sites [19].

**Figure 1:**
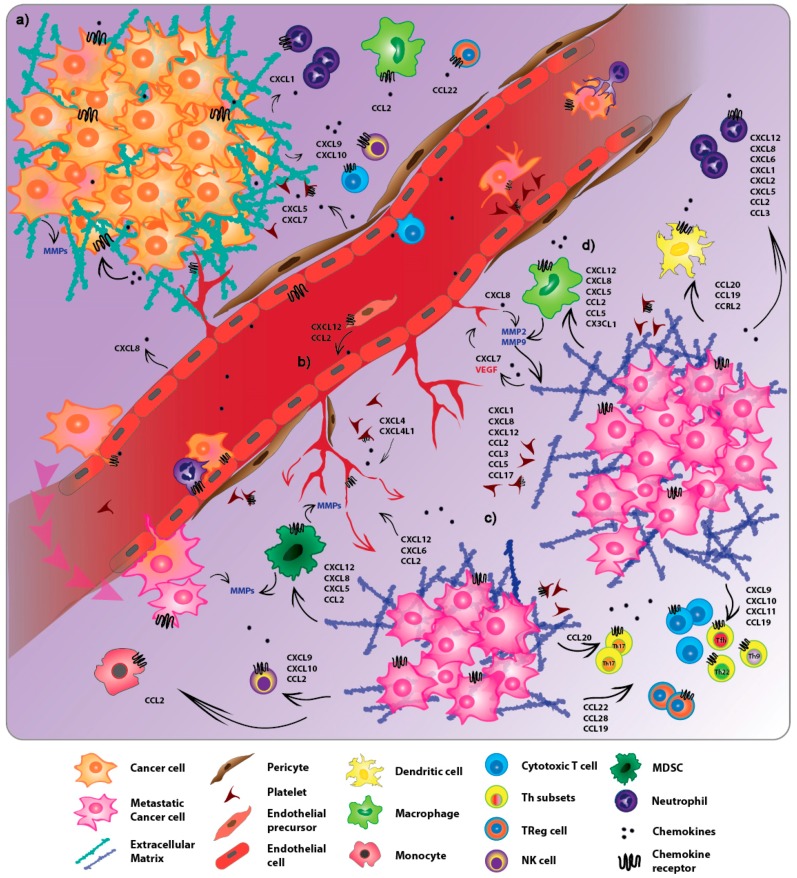
Chemokines contribute to the overall process of tumor metastasis. (**a**) Chemokines promote proliferation and survival of tumor cells at the primary lesion in an autocrine fashion. (**b**) The balance between angiostatic and angiogenic chemokines secreted by stromal cells, immune cells and platelets regulates tumor associated-angiogenesis by supporting endothelial cell proliferation and sprouting, and promoting remodelling of the surrounding extracellular matrix. (**c**) Chemokine gradients shape the tumor microenvironment both in the primary sites and pre-metastatic niches by recruiting different types of immune cells. Whilst T cells and NK cells have an antitumoral effect, T_reg_ cells, tumor associated macrophages (TAMs), myeloid-derived suppressor cells (MDSCs) and tolerogenic dendritic cells (DCs) inhibit antitumor immunity and support the metastatic process. (**d**) Myeloid immune cells accumulating at the pre-metastatic niches, in turn, secrete chemokines that promote angiogenesis and tumor invasion.

In this manuscript, we will focus on the role of different chemokines/chemokine receptors in regulating tumor cell dissemination, angiogenesis as well as tumor immune responses (Figure 1). Additionally, the latest therapeutic strategies targeting chemokine signalling to control tumor progression will be discussed.

## 2. Chemokines and the Tumor Metastatic Behavior

As chemokines contribute to the overall process of cancer growth and metastasis, it is conceivable that a strong correlation between chemokine receptors expression and the clinical outcome of cancer patients could be found [7]. Several studies of human cancer biopsies and mouse models have reported that different tumor types show a peculiar chemokine-receptor profile, and their expression is associated with increased metastatic capacity [9]. 

Here we will provide an accurate overview of the chemokine/receptor pairs that are relevant for tumor metastatization (Table 1). 

The CXC chemokine family plays a key role in promoting survival of cancer cells and guiding metastasis [20]. Among the CXC family, the CXCR4/CXCL12 pair is one of the most investigated chemokine-receptor axes in the metastatic process. Indeed, CXCR4 is commonly expressed in a variety of solid and haematological malignancies including lung, colorectal, gastric, ovarian, prostate and pancreatic cancer, melanoma, esophageal, bladder, head and neck carcinoma, osteosarcoma, neuroblastoma, glioblastoma, and acute lymphoblastic leukaemia [2,20]. It has been shown that the elevated local expression of its ligand CXCL12 guides breast cancer cell trafficking to the lungs, brain, lymph nodes, liver, and bone marrow during the metastatic dissemination. Notably, CXCR4 expression is undetectable in normal mammary epithelial cells [4,7]. In the prostate cancers, high expression of CXCR4 enhances the invasive properties of tumor cells, while low expression levels decrease their metastatic efficiency [21,22]. Interestingly, high levels of CXCR4 have been also observed in CD44^+^/CD133^+^ prostate cancer stem cells (CSC) and are associated with more frequent local recurrence and metastasis formation, suggesting a role of this receptor as a prognostic marker [23,24]. Importantly, CXCR4 also promotes lymph node metastasization from gastric [25] and oesophageal tumors [26], and peritoneal metastasis from ovarian cancers [27]. 

CXCR1 has also been involved in tumor metastasis, as it binds CXCL6 and CXCL8. CXCL8 has a well-established role in mediating initiation and development of various cancers, including breast, prostate and lung cancer, colorectal carcinoma, and melanoma [28]. It has been recently demonstrated that CXCR1 engagement by mesenchymal stem cell-derived IL-8 promotes osteosarcoma cell anoikis resistance and pulmonary metastasis by activating Akt signalling pathway [29]. In addition, it has been found that CXCR1/CXCR2 axis increases melanoma resistance to chemotherapy [30]. 

The CXCR2 receptor binds 7 different ligands (CXCL1, CXCL2, CXCL3, CXCL5, CXCL6, CXCL7 and CXCL8), mainly promoting angiogenesis, invasion, survival and metastasis of colorectal, lung, pancreatic, prostate and renal cancer cells [19]. A vast majority of melanomas express CXCR2, which stimulates metastatic outgrowth, likely due to its ability to bind CXCL8 [31]. However, although CXCL8 protein levels in both tumors and blood sera have been shown to correlate with tumor progression in melanoma patients [32,33], its expression in colon cancer patients has conversely been associated with a significant increase of overall post-operative survival [34]. Notwithstanding, expression of CXCL2, CXCL3 and CXCL8 is increased in colon cancer compared with normal colon tissue and CXCL2 and CXCL3 expression has been found to be already upregulated in premalignant adenomas [34].

CXCR3 receptor also promotes invasion, metastasis and proliferation in melanoma, colorectal cancer and leukemic cells [8,19]. CXCR3 has been found expressed in colon cancer epithelium but has not been detected in normal colonic epithelium [35]. Some human colon cancer cell lines constitutively express CXCR3 and its expression in the primary tumor lesion significantly correlates with lymphatic invasion and lymph node metastasis [36]. Interestingly, although CXCR3 expression correlates with a worse clinical outcome in melanoma, colon cancer, chronic B-cell lymphocytic leukemia, and renal cell carcinoma, reduced expression of CXCR3 in breast cancer has been associated with shorter survival. Intriguingly, as the therapeutic effect of CXCR3 inhibition was compromised in mice depleted of NK cells or with mutations in IFN-γ, it has been suggested that the role of CXCR3 is not simply to mediate tumor cell trafficking but also to support antitumor immunity [37]. 

CXCR5 (mainly expressed by mature recirculating B cells, a small subset of CD4^+^ and CD8^+^ T cells, and skin-derived migratory dendritic cells [38]), and its ligand CXCL13 have been detected in chronic lymphocytic leukaemia and lymphomas. Moreover, the CXCR5/CXCL13 pair is directly involved in the metastatic dissemination of several other tumors, such as pancreatic, colon, and head and neck carcinomas [8,19].

In addition to the CXC chemokine class, the upregulated or aberrant expression of the chemokine receptors of the CC family has been also associated with tumor progression and metastasis. First, the expression of CCR1 that binds to 6 ligands (CCL3, CCL4, CCL5, CCL7, CCL16, CCL23) has been shown to correlate with metastases in lung, prostate, cervical and hepatocellular carcinoma, multiple myeloma, T-cell leukaemia, and osteosarcoma [19,39]. Through exploiting animal tumor models, it has been shown that CCR1 expression increases in colorectal tumor cells during metastasization [40] and that CCR1 sustains liver metastasis by promoting local recruitment of bone marrow (BM)-derived cells that secrete the MMP9 and MMP2 metalloproteinases required for tissue invasion [41]. Furthermore, it has been suggested that, by the dysregulated activation of EGFR signalling, CCR1 may contribute to breast cancer invasion and metastasis [42]. 

It is notable that CCR2, which binds CCL2, as well as CCL7, CCL8 and CCL12, play a controversial role in cancer metastasis. On one hand, CCR2 expression promotes tumor associated macrophages (TAMs) and fibroblast recruitment at the primary tumors, where they enhance invasion, angiogenesis and metastasis of breast, glioma, lung and prostate cancer, melanoma, and multiple myeloma [19]. Importantly, TAMs have been shown to also populate the pre-metastatic sites, where they promote the seeding and establishment of disseminated tumor cells by releasing MMPs [43]. On the other hand, an anti-metastatic activity of the axis CCL2-CCR2 has also been reported, as CCL2 expression levels in prostate cancer correlate with the degree of tumor aggressiveness [44], and absence of CCL2 in cervical cancer has been associated with relapse-free survival [45]. CCL2 levels also significantly increase in acute lymphoblastic leukaemia patients, and further rise during chemotherapy [46].

CCR3, binding 5 different chemokines (CCL5, CCL7, CCL11, CCL24, CCL26), has also been detected in breast, cervical, and renal cells, where it plays a role in regulating the invasion and metastatic process [19]. Furthermore, CCR3 has been implicated in the recruitment and retention of malignant T cells in the skin [77].

CCR4, whose ligands are CCL2, CCL3, CCL5, CCL17 and CCL22 [79], is crucial for immune cell homeostasis but is also involved in hematologic malignancies, such as adult T-cell leukemia and Hodgkin’s lymphomas [80], and some solid tumors, including breast cancer. In breast cancer, CCR4 expression positively correlates with HER2 expression, tumor recurrence, and lymph node, lung, and bone metastasis, as it enhances chemotaxis to CCL17. Thus, it is not surprising that a strong correlation between CCR4 expression and lower overall survival and disease-free survival was reported in breast cancer patients [81]. CCR4 also drives melanoma cells to metastasize in the brain [84] and is associated with metastases and poor prognosis in hepatocellular carcinoma patients, as it increases epithelial-mesenchymal transition (EMT) and MMP2 expression [85]. 

CCR5 receptor, that binds CCL3, CCL4, CCL5 and CCL8, is also involved in TAMs recruitment and plays a role in guiding invasion and metastasis of breast, cervical, lung, multiple myeloma, osteosarcoma, pancreatic, and prostate malignancies [19,86]. 

CCR6 has been found in colorectal, breast, and hepatocellular carcinomas [19], and its expression is associated with colorectal cancer progression, metastasis, worse staging and poor overall survival of colon cancer patients [89]. Additionally, CCR6 is overexpressed in liver metastases, as it supports cancer cell migration towards secondary sites in thyroid, ovarian and colon carcinomas [90]. The upregulation of both CCR6 and its ligand CCL20 is also observed in metastasis of advanced cutaneous T-cell lymphoma [111] and it has been involved in the development and progression of laryngeal cancer [92]. In line with the reports showing that CCL20 induces proliferation of cultured human breast epithelial cells, CCR6 expression has been correlated with poor clinical outcome and decreased relapse-free survival of breast cancer patients [93].

CCR7, CCR9, and CCR10 were also described to directly support metastatic seeding of cancer cells [2]. CCR7 receptor activity is fundamental for immune cell entry into lymphatic vessels and it binds the homeostatic chemokines CCL19 and CCL21, preferentially expressed in secondary lymphoid tissues. As it supports cell trafficking to the lymphatics, CCR7 has been identified primarily in lymph node metastases and high levels of CCL21 and CCL19 have been found in the draining lymph nodes of different tumors [7,9]. CCR7 is in fact expressed in breast, gastric, colorectal, non-small-cell lung, oesophageal squamous cancer, and chronic lymphocytic leukemia [4,94,96,101,112], and its expression correlates with metastatic potential and poor prognosis [9]. 

CCR9 has been reported to promote melanoma metastasis to the small intestine [97]. Clinical studies showed that CCR9-expressing human melanomas display a very high probability of metastasizing to the small intestine, expressing a high level of the CCR9-ligand CCL25 [97]. Moreover, CCR9 over-expression has been reported to support prostate cancer cell invasion and metastasis by modulating their MMPs expression [98].

Human melanoma cells also express CCR10, which binds to CCL27 and CCL28; these chemokines are expressed at high levels in skin, which is a common site of metastasis from different tumors [99]. CCR10 has also been involved in cancer growth and invasion in adult T-cell leukaemia/lymphoma and chronic lymphocytic leukaemia [113,114]. 

CCR8 also supports cancer dissemination, as its ligand CCL1, produced by lymphatic endothelial cells in the subcapsular sinus, can attract CCR8^+^ tumor cells to the lymph nodes to promote metastasis [102].

The chemokine CX3CL1 and its specific receptor CX3CR1 are also significantly upregulated in tumors, and their expression has been associated with increased metastatic capacity in bones [103]. Moreover, it has been demonstrated that CX3CR1 overexpression is an early event in pancreatic cancer progression and correlates with tumor invasion of local nerves and ganglia [105,106]. The intrapancreatic and extrapancreatic nerves are often the target of pancreatic ductal adenocarcinomas because of their secretion of CX3CL1 that generates a gradient supporting cancer cell migration [106]. Despite the fact that in prostate cancer CX3CR1 expression correlates with bone metastasis, its expression in breast cancer predicts occurrence of brain metastasis [115,116].

Intriguingly, atypical chemokine receptors (ACKRs) have been largely demonstrated to play a role in cancer biology. In contrast to canonical chemokine receptors, ACKRs are mainly expressed by non-leukocyte cell types, such as erythrocytes and lymphatic or vascular endothelial cells, and have not been considered capable of inducing cell migration [117]. ACKRs bind chemokines with high affinity but are unable to couple to G-proteins, and their activation generally triggers β-arrestin-dependent pathways. ACKRs regulate chemokine bioavailability by enhancing chemokine/receptor degradation or relocalization in polarized cells and regulate the expression and signalling of other canonical chemokine receptors [118]. Notably, the expression of ACKRs has been reported to be deregulated in cancer cells and tumor specimens and correlate with metastatic potential [119].

ACKR1, previously known as Duffy antigen receptor for chemokines (DARC), is a promiscuous chemokine receptor binding CXCL1, CXCL2, CXCL3, CXCL5, CXCL6, CXCL7, CXCL8, and some CC chemokines [120]. ACKR1 acts as a ‘‘decoy” for excess chemokine and has been suggested to limit tumor metastasis by dampening the pro-angiogenic environment. Indeed, overexpression of ACKR1 decreases tumor cellularity, vascularity and metastasis in different tumor mouse models, such as in breast cancer, where it significantly inhibits tumorigenesis and lung metastasis. [121]. Beyond ACKR1, the atypical receptor ACKR2, previously called D6, has been detected in tumor specimens, where it plays a negative role in tumor growth and metastasis [117]. ACKR2 binds inflammatory CC chemokines, and is primarily expressed by lymphatic endothelial cells, trophoblasts and some leukocyte subsets. Importantly, it has been demonstrated that ACKR2 prevents the development of skin tumors by chemokine sequestration and its expression positively correlates to disease-free survival rate in cancer patients [122]. However, it has been recently demonstrated that ACKR2 also hinders tumor infiltration of NK cells by limiting their expression of CCR2, finally supporting metastasis [107].

ACKR3, also known as CXCR7, is another atypical receptor binding CXCL12 and CXCL11 [109], and has been shown to regulate breast cancer metastases. ACKR3 is expressed in many tumor cell lines and its expression is also elevated in endothelial cells associated with tumors [123]. Importantly, ACKR3 staining of high-density tissue microarrays demonstrated that its expression at the protein level is elevated in aggressive prostate tumors. ACKR3, in turn, increases the expression of pro-angiogenic factors such as IL-8 and VEGF [124] and supports transendothelial migration of cancer cells [125].

ACKR4/CCRL1 binds the homeostatic chemokines CCL19, CCL21, CCL25, and CXCL13 and is expressed by thymic epithelial cells, bronchial cells, and keratinocytes [126]. ACKR4 is less well-characterized in comparison with the other ACKRs. However, as its ligands are involved in cancer development and metastasis, it can be speculated that it might play a role in modulating the metastatic process [127].

Finally, ACKR5/CCRL2, initially described to promote chemotaxis in response to CCL2, CCL5, CCL7, and CCL8 [128], has also been shown to be involved in tumor metastasization, as its expression has been detected in colorectal cancers and even increases in the early phase of liver colonization [40].

Thus, as chemokine/chemokine receptor expression in tumors significantly correlates with metastasis recurrence and clinical response to therapy [20], the development of reliable and cost-effective assays for the routine screening and follow-up of cancer patients might provide a powerful diagnostic and prognostic tool to improve clinical outcome. 

## 3. Chemokine Axes and Tumor Angiogenesis

The development of new blood vessels, ensuring adequate oxygen and nutrients delivery and clearance of metabolic wastes, is essential for tumor cell survival [129]. This process, named angiogenesis and consisting of the formation of new branches from a pre-existing capillary network [130], is a finely regulated process representing a rate-limiting step for tumor growth [131].

Chemokines are essential elements for the regulation of tumor-associated angiogenesis [7,132], as they can mediate signals promoting endothelial cell proliferation and sprouting, but they can also activate angiogenic inhibitory pathways [133,134]. 

Angiogenic CXC chemokines include CXCL1, CXCL2, CXCL3, CXCL5, CXCL6, CXCL7, CXCL8, and CXCL12. They mainly provide the stimulatory signals through the interaction with CXCR2 and CXCR4, this latter uniquely for CXCL12 [7,135,136]. Conversely, angiostatic CXC chemokines, among them CXCL4, CXCL9, CXCL10, and CXCL1, inhibit the neo-angiogenesis process, usually binding CXCR3-B [136]. 

CXCL8/IL8, secreted primarily by endothelial cells, has been shown to be a key mediator of angiogenesis in different tumors [137,138,139,140]. Its expression is triggered by multiple stimuli, including cytokines (interleukin-1, interleukin-6, CXCL12, and TNFα), hypoxia, reactive oxygen species (ROS), pathogen associated molecular patterns (PAMPs) and other environmental stresses [141]. IL8 directly supports endothelial cell survival and proliferation by causing the over-expression of the antiapoptotic Bcl-2 protein [142,143] and prompting the release of the vascular endothelial growth factor (VEGF) from the endothelium [144]. Furthermore, an autocrine IL8-dependent signalling enhances MMP-2 and MMP-9 production in endothelial cells, allowing extracellular matrix (ECM) remodelling that is necessary to form new blood vessels [145]. 

Notably, the tumor stromal compartment can, in turn, modulate chemokine activity, as for CXCL7/NAP-2 and CXCL12. CXCL7/NAP-2 can support cancer cell proliferation and the expansion of the tumor-associated lymphatic network, by directly controlling the expression of VEGF-C/D and heparanase [146]. Furthermore, the maturation of tumor-released CXCL7/NAP-2, whose expression is enhanced by IL1-beta [147], is regulated by proteolitic cleavage mediated by MMPs [148] or cathepsin G, expressed by neutrophils [149]. 

Interestingly, the expression of MMPs, and in particular the release of pro-MMP-9, is promoted by tumor-derived CXCL12 in an autocrine fashion [150]. During hypoxia and upon angiogenic factor stimulation [50], CXCL12 can be secreted by specialized stromal cells [51], thus exerting multiple effects on both cancer and endothelial cells. When CXCL12 accumulates within the tumor microenvironment, endothelial cell migration, proliferation, tube formation, and VEGF secretion are all enhanced [151]. Moreover, CXCL12 gradient fosters endothelial activation by increasing the expression of the intercellular adhesion molecule-1 (ICAM-1) [152]. Interestingly, both cancer and endothelial cells express the atypical receptor ACKR3/CXCR7 that regulates cell invasion, adhesion and angiogenesis [108,153], through the activation of the Akt-dependent pathway [154].

It has been reported that the intratumoral accumulation of CCL2 promotes the homing of endothelial progenitor cells, thus supporting the neovascularization process [155]. CCR2, the CCL2 receptor, is in fact expressed by endothelial cells and, upon ligand binding, induces endothelial cell chemotaxis and tube formation [156] through the up-regulation of the transcription factors Ets-1 and MCPIP [157,158] and the expression of VEGF-A [159]. 

CXCL6, also named granulocyte chemotactic protein-2 (CXCL6/GCP-2), is another chemokine involved in the regulation of tumor angiogenesis. CXCL6 secretion is predominantly induced by IL-1β stimulation of mesenchymal cells, including fibroblasts and macrovascular endothelial cells, as a result of chemokine NH_2_-terminal truncation [160,161]. Once released within the tumor, CXCL6/GCP-2 acts both as a growth factor inducing endothelial [162] and cancer cell proliferation [161] and as chemoattractant agent for neutrophils and endothelial cells [162,163,164]. Intratumoral CXCL2 also attracts granulocytes, that in turn, boost cancer cell survival [165] and angiogenesis, by releasing additional chemokines [135,166]. Remarkably, the stromal and tumor expression of CXCL2, together with CXCL1, is regulated by the exposure to pro-inflammatory cytokines, such as TNF-alpha [56]. Recent publications showed that CXCL1/CXCL2 chemokines, together with CXCL5, cooperate in the recruitment of a specific Gr-1^+^CD11b^+^ myeloid population, called myeloid-derived suppressor cells (MDSCs) [167,168,169]. Within the tumor, MDSCs contribute to vasculature formation, mainly by the secretion of pro-angiogenic factors [170,171,172]. In addition to its crucial involvement in MDSC recruitment, it has been reported that CXCL5 overexpression in non-small cell lung cancer (NSCLC) enhances both tumor growth and angiogenesis by a cyclooxygenase (COX-2) dependent mechanism, whereas its depletion attenuates this phenotype [173,174]. Alterations in the COX-2 activity lead to an accumulation of its major metabolic product, prostaglandin E2 (PGE_2_), that has a crucial role in cancer development [175]. PGE_2_ controls the local recruitment of MDSCs by regulating their expression of CXCL12 and CXCR4 [176] and stimulates tumoral secretion of the pro-angiogenic CXCL1, thus promoting micro-vessel formation and cancer progression [177].

As previously mentioned, the tumor milieu also houses angiostatic chemokines, inhibiting tumor-associated angiogenesis primarily by binding CXCR3 [133] expressed on the micro-vasculature [178,179]. Local accumulation of the pro-inflammatory IFN-γ stimulates the recruitment of CXCR3-positive cells and promotes the release of angiostatic chemokines, among them CXCL9, CXCL10, and CXCL11 [180]. These chemokines, as well as CXCL4, CXCL4L1, and CXCL14, have been reported to inhibit angiogenesis, dampening tumor growth and metastasis [181,182,183,184,185,186].

CXCL4 and CXCL4L1 are mainly released by activated platelets [52]. As platelet α-granules contain both pro-angiogenic and angiostatic factors, platelets are strongly involved in tumor angiogenesis [63]. Platelet factor-4 (CXCL4/PF-4) was the first chemokine shown to inhibit angiogenesis. However, CXCL4L1/PF-4var, later isolated from thrombin-stimulated platelets and differing from CXCL4/PF-4 in three carboxy-terminal amino acids, shows higher anti-angiogenic activity and stronger ability to inhibit tumor growth than CXCL4/PF-4 [52].

However, platelets have also been demonstrated to promote the survival of circulating tumor cells (CTCs) in the bloodstream by conferring their resistance to shear stress and attack from NK cells [64]. Within the tumor microenvironment, platelets secrete CXCL5 and CXCL7, as a consequence of direct contact with tumor cells [57] or following activation of the coagulation cascade [58], thus supporting the recruitment of CXCR2-positive myeloid cells [59]. Platelet granules contain several other chemokines, including CXCL1, CXCL8/IL-8, CXCL12, CCL2, CCL3, CCL5, and CCL17 [52]. The simultaneous release of pro-inflammatory factors from platelets and tumor cells themselves attracts a variety of immune cells to form tumor cell–platelet emboli [57], strongly supporting the metastatic process [187].

Notably, platelets were also found to promote epithelial to mesenchymal transition (EMT) on CTCs through the secretion of TGFβ. Platelet-derived growth factors confer a mesenchymal-like phenotype to tumor cells and open the capillary endothelium to support extravasation in distant organs. It has been recently shown that, by releasing Lysophosphatidic acid (LPA), known to induce tumor invasiveness via proximal CD97-LPAR heterodimer signaling, platelets support extravasation and establishment of metastatic cells in distant organs [87].

A deep understanding of the mechanisms regulating the fine equilibrium between pro-angiogenic and anti-angiogenic chemokines secreted by different cells accumulated within the tumors might allow the development of more powerful strategies to complement the conventional cancer therapy and prevent metastatic tumor dissemination. 

## 4. Chemokines and Tumor Immunity

Tumors are crowded lesions where different numbers and types of non-malignant cells, such as stromal cells and immune cells, come running to either support or prevent tumor immunity and metastasization. In addition to their direct effect on tumor cells themselves and the endothelium, chemokines play a fundamental role in immune cell recruitment and infiltration into tumors, thus modulating antitumor immune response and cancer cell dissemination. 

A comprehensive meta-analysis of more than 5000 tumor specimens from patients with breast, colorectal, lung, ovary, head and neck carcinomas, as well as melanomas, recently revealed that a universal pattern of correlation exists between the relative abundance of distinct leukocyte subsets infiltrating the tumors and the expression of individual chemokines-receptors, regardless of the cancer type and localization [78]. Indeed, CCR1, CCR2, CCR5, CXCR4, CCL18, CCL19, CCL21, and CXCL12 expression has been identified to positively correlate with immune infiltration of breast and colorectal carcinomas, non-small cell lung cancer, and melanomas [78].

The homeostatic chemokines CXCL12 and CCL21 have been reported to be crucial players in supporting all the stages of the metastatic process, as they physiologically dictate cell positioning throughout the body, both during tissue development and immune response [100]. Importantly, the aforementioned meta-analysis revealed that tumor infiltration of T cells and neutrophils are mutually exclusive, as neutrophil recruitment inversely correlates with CXCL9, CXCL10 and CXCL11, which conversely promote lymphocyte accumulation [78].

It has been reported in both mouse models and human samples that antitumoral effector CD8^+^ T cells, Th1 cells, and NK cells migrate into tumors, thanks to their expression of CXCR3 receptor. High levels of its ligands CXCL9 and CXCL10 have been associated with decreased levels of cancer metastasis and improved survival in patients with ovarian cancer and colon cancer [61,62]. Notably, other different subsets of lymphocytes reach the tumor microenvironment to modulate tumor immune responses in both primary tumors and metastatic sites. Th_17_ cells, mostly mediating potent antitumor immunity by recruiting CD8^+^ T cells [82], NK cells [83], and dendritic cells (DCs) [188] into the tumor microenvironment, express high levels of CCR6 and CXCR4 and low levels of CCR7 [49]. Thus, as the CCR6 ligand CCL20 has been found in human tumor samples [54], it has been suggested that it facilitates the recruitment of antitumoral Th_17_ into tumors. However, it seems that Th_17_ are less prone to home to lymphoid tissues because of the absence of CCR7 [13]. Notwithstanding, Th_22_ cells, expressing high levels of CCR7, support tumor progression and have been shown to infiltrate colon and pancreatic cancers and hepatocellular carcinomas [82]. Regulatory T cells (T_reg_), known to suppress antitumor immunity, express CCR4 [55] and CCR10 [189], by which they are recruited into the tumor microenvironment. T_reg_ cells reach the tumor lesions by sensing CCL22, mainly produced by tumor associated macrophages (TAMs) and cancer cells [190], and CCL28, found in tumor hypoxic regions [189]. Notably, IL17^+^ regulatory T cells can express CXCL8 in human colon cancer microenvironment, thus promoting inflammation and cancer cell dissemination [191].

In addition, stromal cells and endothelial cells embedded into the growing tumors, as well as macrophages, dendritic cells, and neutrophils, secrete a multitude of chemokines that support cancer cell survival, angiogenesis and migration towards pre-metastatic sites [7].

In recent decades, multiple chemokine/chemokine receptor pairs, as CCL2/CCR2, CCL5/CCR5/, CXCL5/CXCR2, and CXCL12/CXCR4, have been reported to indirectly promote tumor progression by increasing the recruitment and suppressive activity of tumor associated macrophages (TAMs) and myeloid-derived suppressor cells (MDSCs) [192,193]. Importantly, besides regulating the equilibrium between antitumor immunity and immune tolerance at the primary sites, myeloid immune cells participate to the processes of cancer cell invasion and dissemination. Growing evidence has been in fact shown that neutrophils, macrophages and dendritic cells may actively support the metastatic process [194].

Neutrophils are recruited at the tumor sites by several chemokine signals, such as CXCL12, CXCL8, CCL3 (MIP-1α), and CXCL6 (huGCP-2), or murine chemokines CXCL1, CXCL2, and CXCL5 [195]. It has been reported that, thanks to CXCR2 engagement, they reach the pre-metastatic niches in colorectal cancer [196] and metastatic pancreatic cancer models [197]. Circulating neutrophils promote metastasis by entrapping cancer cells into their neutrophil extracellular traps (NETs), by which they promote tumor cell early adhesion at distant organ sites. [65] NET-like structures have been shown to surround breast tumor cells in both mouse models and clinical samples of breast cancers [66]. Intriguingly, it has also been recently demonstrated that NETs awaken dormant cancer by concentrating neutrophil proteases at the ECM, promoting tissue remodelling and integrin-mediated signalling in cancer cells [67]. Neutrophils also support cancer cell survival in the circulation [68] and cell seeding at the pre-metastatic niche by facilitating cancer cell adhesion to the endothelium by CD11b and selectin expression [69,70]. Additionally, CCR1 expressing neutrophils might be recruited into liver metastatic foci by CCL9 [41], and CCR2 positive neutrophils have been identified in pre-metastatic lungs [41], where they specifically sustain metastatic initiation [71]. Importantly, CCL2 signalling increases the cytotoxicity of neutrophils against murine and human breast cancer models, thus suggesting that it might trigger neutrophil anti-metastatic capacity [72].

Besides neutrophils, intra-epithelial macrophages, recruited in the tumor microenvironment thanks to chemokine sensing, have been shown to favor early stage breast cancer dissemination by supporting E-cadherin disruption [73]. CCL2 might be considered the main attractant for macrophages and MDSCs into the tumor microenvironment and triggers macrophage mediated angiogenic switch [74,75] and MDSC-dependent suppression of antitumor immunity [47,76]. CCL2 also recruits inflammatory monocytes [104] to pre-metastatic sites and mediates macrophage differentiation and polarization [95]. It has been in fact shown that it promotes macrophage differentiation towards pro-tumoral alternatively activated M2-type macrophages [60], via inhibition of caspase-8 cleavage and enhanced autophagy [198]. Notably, differently from classical activated M1 macrophages that exert tumoricidal activity, M2 macrophages promote tumor growth and survival, angiogenesis, and metastasis [48]. Similarly, CCL2 has been reported to regulate T cell differentiation and polarization [199], promoting Th2 polarization towards a more immunosuppressive T regulatory phenotype in vitro and in mouse models [200]. 

CCL2-CCR2 axis has been reported to enhance TAMs secretion of CCL3 at the pre-metastatic sites, thus sustaining lung metastasis seeding in mouse models of breast cancer [53]. CCL5 expression in breast malignancies has been also associated with macrophage infiltration and prognosis. Similarly to CCL2, CCL5 promotes the occurrence of deleterious TAMs and inhibits potential antitumor T cell activities, favoring the metastatic process of breast cancers [201]. In addition to this, CXC3L1 (originally named Fractalkine)-CXC3CR1 axis crucially contributes to the pro-metastatic activity of TAMs. In fact, despite the fact that CX3CL1 expression does not correlate with the density of tumor-infiltrating macrophages [103], it has been shown that it promotes macrophage survival in tumor microenvironment contributing to tumor cell dissemination [202]. Further, CX3CR1 deficiency increases the apoptosis of pro-angiogenic macrophages in mouse models of colon cancer, thus inhibiting liver metastases formation [202]. 

In response to IL-4, IL-13 and IL-10, TAMs produce CCL18, which has been shown to promote breast tumor progression and metastasis both in preclinical models and in breast cancer patients [203]. High levels of CCL18, both the intratumoral and the circulating ones, were indeed associated with a worse prognosis of cancer patients [203]. Moreover, TAMs secrete high levels of CXCL1, which promotes EMT of breast cancer cells and lymph node metastasis via activating NF-κB/ SOX4 signalling [204].

MDSCs are part of the immunosuppressive components in tumor-bearing hosts. They represent a heterogeneous population of myeloid cells that includes both mature and immature subsets of monocytic, granulocytic, and dendritic cells, preventing antitumor immune response through different mechanisms, such as essential metabolite consumption, reactive oxygen (ROS) and nitrogen species (RNS) production, and generation of T-cell trafficking dysfunctions [91]. Notably, both the CXCL5–CXCR2 and CXCL12–CXCR4 signalling pathways promote MDSC trafficking in tumor microenvironment [205]. MDSCs accumulation in both primary solid tumors and metastatic sites correlates with the overall survival, as well as the disease-free survival, response to chemotherapy, and metastatic progression in cancer patients [206]. It has been demonstrated that CCR5 axis plays a key role in driving MDSCs development within the bone marrow and promoting their migratory and immunosuppressive properties to support tumor development [110]. CCL2 is also a strong chemoattractant molecule for MDSCs, even when it is post-translationally modified by RNS [47]. Furthermore, CXCL8 secreted by tumor and T_reg_ cells has been shown to sustain MDSC migration and degranulation via CXCR1 and CXCR2 signalling to support cancer dissemination [207]. It is notable that MDSCs infiltrating primary tumor lesions by sensing CXCL1, CXCL2, and CXCL5 promote epithelial-mesenchymal transition (EMT) and dissemination of cancer cells, by secreting TGF-β, EGF, and HGF [208]. 

Intriguingly, although mature DCs, that can drive potent antitumor immune responses by priming and activating tumor antigen-specific T cells, have not been found to accumulate at tumor lesions, [209] immature DCs, that rather induce peripheral tolerance by promoting the expansion of T_reg_ cells [210], have been demonstrated to infiltrate melanomas, hepatomas, prostate, lung, breast and gastric cancers, renal cell carcinomas, and neuroblastomas [88]. In fact, while immature DCs, expressing CCR6, follow CCL20 gradients generated by tumors themselves, mature DCs express CCR7, that is required to migrate towards CCL19 and CCL21, key players in homeostatic formation of secondary lymphoid structures. Both CCL21, produced by high endothelial venules, lymphatic endothelial cells, and intranodal fibroblastic reticular cells, and CCL19, secreted by fibroblastic reticular cells within the lymph nodes, recruit T cells and T_reg_ cells, B cells, and mature DCs to initial lymphatics and lymph nodes to elicit immune response [211]. Therefore, it is not surprising that the vast majority of DCs found in the tumor microenvironment are in their immature state and thus unable to trigger immune responses. However, it has also been demonstrated that early metastases from pancreatic ductal carcinomas are associated with dense networks of an immunosuppressive dendritic cell subset, whose depletion causes cytotoxic lymphocyte activation, T_reg_ cells reduction and metastasis inhibition [212].

Additionally, the recently identified CXCL14 can modulate tumor immune cell infiltration. Although the receptor for CXCL14 is still unknown, it has been reported that CXCL14 is able to synergize with CXCL12 via allosteric modulation of CXCR4 [213], and recruit myeloid DCs to the tumor sites, where it likely promotes their maturation to sustain antitumor immunity [214]. Indeed, it has been recently reported that CXCL14/BRAK transgenic mice display a significant lower rate of induced colorectal carcinogenesis, decreased tumor size when injected with tumor cells, and reduced number of lung metastatic nodules in comparison with wild-type mice [215].

Because of the high complexity of the tumor microenvironment, a deeper knowledge of the mechanisms regulating chemokine sensing of the different cell types, both immune/stromal and malignant cells, within the tumor lesions and the pre-metastatic niches seems to be crucial for preventing tumor metastasization. 

## 5. Chemokine Targeting in Cancer Therapy

Chemokines have been described as key regulators of the metastatic spread, as they may facilitate the tumor cell survival, proliferation, and invasiveness [31,216], and are thought to directly drive metastatic cells to target organs [9]. Given that, the control of different chemokine/chemokine receptor axes has been proposed and exploited as an attractive therapeutic strategy for cancer treatment. For instance, Mogamulizumab, a humanized monoclonal antibody directed against the CCR4 receptor, has been approved for the treatment of adult T-cell leukemia [217] and, more recently, for skin lymphoma (Poteligeo) [218].

The primary tumor microenvironment has represented an attractive site for chemokine-oriented therapies, since it acts as the scenario where cancer and immune cells first interact [13]. Years of investigations have attempted to draw a fine line within the chemokine system discriminating factors that can either promote or suppress tumor development. A plethora of pre-clinical studies have been designed and developed in multiple tumor mouse models. This issue has been authoritatively discussed in seminal reviews [7]. 

The chemokine CXCL12 and its receptors, CXCR4 and CXCR7, participate in tumorigenesis and metastasis in several types of cancers [219]. It has been reported that the overexpression of the chemokine receptor CXCR4 in melanoma cells is sufficient to promote metastatic accumulation of tumor cells in lungs, and pharmacological blocking of CXCR4 by a specific inhibitor prevents in vivo pulmonary metastases in CXCR4-B16 melanoma-bearing mice [220]. Notwithstanding, combinatorial approaches may even progress the efficacy of chemokine-based therapy. Indeed, the combination of the small CXCR4 antagonist, AMD3100, with the multikinase inhibitor sorafenib and anti-PD-1 treatment enhanced CD8 T cell mediated antitumor immunity in a mouse model of advanced hepatocellular carcinoma (HCC) [221]. The blockade of the CXCR4 pathway during sorafenib treatment inhibits lung metastasis and improves the overall survival by preventing tumor vascular growth and increase of EMT markers. Moreover, AMD3100 boosts the efficacy of anti-PD1 treatment by triggering tumor death and decreasing metastasis incidence. Finally, the use of the triade sorafenib/AMD3100/anti-PD-1 promotes the repositioning and activation of intra-tumoral CD8^+^ T-lymphocytes [221]. 

The CCL2/CCR2 pair actively participates in tumor progression and metastasis: high serum levels of this chemokine correlated with poor prognosis in breast carcinoma patients [222]. Furthermore, CCL2 may directly promote tumor cell migration and, remarkably, may favor the extravasation and seeding of breast cancer cells through the recruitment of inflammatory monocytes to the pre-metastatic foci [104]. Based on a strong preclinical rationale [104], a humanized antibody against CCL2, carlumab, has been developed and tested in the first-in-human, first-in-class, phase I study in patients with solid malignancies [223]. Regrettably, results obtained using tumor mouse models were not transferable to patients. Authors attributed the low level of clinical effectiveness of therapy to several reasons, mainly chemokine promiscuity and to important differences between the mouse MCP-1 and human CCL2. Importantly, a relevant issue that must be discussed in this regard is the pivotal role of CCL2 in early tumor immune-surveillance. In fact, although the growth of primary mammary carcinoma was significantly delayed in *Ccl2^−/−^* or *Ccr2^−/−^* mice, the number of spontaneous pulmonary metastases increased in the same tumor-bearing hosts [224]. Thus, if on one hand, the abrogation of CCL2 signalling may prevent tumor cell dissemination, on the other hand, it can also impair the recruitment of antitumor immune cell subsets that actively counteract cancer growth and spreading. Thus, as for other chemokines, a big effort needs to be made to define the timing and dosage of CCL2-inhibition in tumor-bearing hosts. 

Importantly, despite the fact that several small molecule inhibitors and antibodies specifically targeting a single chemokine-chemokine receptor axis successfully repressed cancer growth in animal models, their translation to the clinic as mono-therapy remains unsatisfactory. This discrepancy can be attributed to various reasons, primarily the old concept of “chemokine redundancy”; furthermore, it may reflect important biological differences between mouse and human chemokines. While revising this matter in the context of inflammatory and autoimmune diseases, Shall and Proudfoot pointed out the inappropriate target selection and the insufficient in vivo dosing of chemokine receptor blockers as the main clinical hurdles for improving therapies [225]. On the other hand, they manifestly declined the idea of the redundancy of the chemokine system, rather proposing that temporal and spatial control of different chemokine signals in vivo determines different biological outcomes in different tissues [225]. In line with this, subsequent reports characterizing small molecule agonists for the CXCR3 receptor further substantiated the notion that “molecular redundancy” is not a feature of the chemokine system [226,227]. 

Hence, although the therapeutic exploitation of chemokine/chemokine receptor axes still represents a feasible option, further studies are required to improve tools and strategies that are currently available in the field. Combinatorial approaches exploiting chemokine/chemokine receptor targeting, standard care treatments (chemo-radio- therapy), and new immunotherapeutic tools (mAb; CAR T and check-point inhibitors) might represent conceivable solutions to improve the therapeutic outcomes in cancer patients. 

Therefore, it is imperative to better characterize chemokine/chemokine receptor identity and functions in cancer. An in-depth analysis of when and how a single (or multiple) chemokines and chemokine receptors contribute to tumor development and spreading is pivotal for the definition of novel therapeutic approaches that can be successfully translated to the clinic. 

## 6. Conclusions

Chemokine and chemokine receptors are intrinsic features of the tumor landscape, characterizing both primary tumor lesions and metastatic sites. Notably, besides their traditional well-known functions in directing cell migration, chemokines also play critical roles in tumor initiation, promotion and progression [7,8]. Within the tumor microenvironment, as reviewed in this manuscript, distinct chemokine/chemokine receptor pairs control cancer cell survival and/or growth, but they also modulate angiogenesis and cancer cell dissemination, shape the pre-metastatic niches, and, importantly, influence antitumor immunity. Chemokines can be detected at the tumor primary lesions, in the blood sera, and at the pre-metastatic niches in both preclinical models and human patients. Intriguingly, all the different cell types crowding the tumor microenvironment release chemokines. It has in fact been demonstrated that tumor cells themselves, endothelial cells, platelets and different types of immune cells, belonging to both the myeloid or lymphoid compartment, release chemokines and express chemokine receptors. Thus, it is not surprising that a strong correlation between chemokine/chemokine receptor expression and the clinical outcome of cancer patients has been found [7]. Because of this, the targeting of distinct chemokine axes has been proposed and tested as a therapeutic strategy to counteract cancer growth and spreading.

However, despite encouraging results, the clinical exploitation of chemokine-based therapies has been less successful than expected and further investigations are still necessary to increase their efficiency. As chemokines also regulate immune cell trafficking within the tumors, a better characterization of chemokine/chemokine receptor identity and functions in the whole tumor microenvironment might be crucial to understand the effect of one chemokine on different cell types infiltrating the tumors and the pre-metastatic niches at different stages. A deep knowledge of the spatial and temporal effect of the same chemokine on different cell subsets at the pre-metastatic niches might allow the design of tunable strategies to concomitantly prevent cancer cell dissemination and stimulate antitumor immunity. 

In this way, the development of combined approaches targeting chemokine axes in patients undergoing conventional radio- or chemotherapy with antitumor immunotherapy might represent a very promising strategy to improve the clinical outcomes of cancer patients.

## Figures and Tables

**Table 1:** Chemokines and chemokine receptors contributing to tumor metastasis. Chemokine and chemokine receptor pairs identified in cancer lesions and promoting tumor growth and stromal/immune cell recruitment. Question marks indicate that the respective chemokine receptor is currently unknown.

Chemokine Receptor	Ligand(s)	Type of Cancer Cells	Type of Stromal/ Immune Cells
CXCR4	CXCL12	Breast cancer [3,7]	TAMs [47]
		Prostate cancer [20,21,22,23]	MDSCs [47,48]
		Gastric cancer [24]	Neutrophils [49]
		Esophageal cancer [25]	Endothelial cells [50]
		Ovarian cancer [26]	Precursors of endothelial cells [51]
			Platelets [52]
CXCR1	CXCL6 CXCL8	Breast cancer [27]	Neutrophils [49]
		Prostate cancer [27]	MDSCs [53]
		Lung cancer [27,28]	
		Colorectal cancer [27]	
		Melanoma [27]	
CXCR2	CXCL1 CXCL2 CXCL3 CXCL5 CXCL6 CXCL7 CXCL8	Colorectal cancer [18,33]	Neutrophils [49,54,55]
		Lung cancer [18]	MDSCs [48,56]
		Pancreatic cancer [18]	Platelets [52,57,58,59]
		Prostate cancer [18]	
		Renal cancer [18]	
		Melanoma [30,31,32]	
		Breast cancer [60]	
CXCR3	CXCL9 CXCL10 CXCL11 CXCL4L1	Melanoma [18,34]	T cells [61,62]
		Colorectal cancer [8,18,34,35]	NKT cells [63,64]
		Leukemia [18,34]	Platelets [52]
		Breast cancer [36]	
		Renal cancer [18,34]	
CXCR5	CXCL13	Lymphomas [18,34]	
		Pacreatic cancer [18,34]	
		Colon cancer [18,34]	
		Head and neck carcinomas [18,34]	
CCR1	CCL3 CCL4 CCL5 CCL7 CCL14 CCL15 CCL16 CCL23	Lung cancer [18]	Neutrophils [40,49]
		Prostate cancer [18]	
		Cervical cancer [18]	
		Hepatocellular carcinoma [18]	
		Multiple myeloma [18]	
		T cell leukemia [18]	
		Osteosarcoma [38]	
		Colorectal cancer [39]	
		Breast cancer [41]	
CCR2	CCL2 CCL7 CCL8 CCL12 CCL13	Breast cancer [18]	TAMs [47,65,66,67,68,69,70,71,72,73,74,75,76]
		Glioma [18]	MDSCs [67,68]
		Lung cancer [18]	Monocytes [69]
		Prostate cancer [18]	Platelets [52]
		Melanoma [18]	
		Multiple myeloma [18]	
CCR3	CCL5 CCL7 CCL11 CCL13 CCL15 CCL24 CCL26 CCL28	Breast cancer [18]	Platelets [52]
		Cervical cancer [18]	
		Renal cancer [18]	
CCR4	CCL2 CCL3 CCL5 CCL17 CCL22	T cell leukemia [77]	T_reg_ cells [78]
		Hodgkin lymphoma [77]	Monocytes [69]
		Breast cancer [79]	Platelets [52]
		Melanoma [80]	
		Hepatocellular carcinoma [81]	
CCR5	CCL3 CCL4 CCL5 CCL8	Breast cancer [18,76]	TAMs [47,82,83]
		Cervical cancer [18]	
		Lung cancer [18]	
		Multiple myeloma [18]	
		Osteosarcoma [84]	
		Pancreatic cancer [18]	
		Prostate cancer [18]	
CCR6	CCL20	Colorectal cancer [18,85,86]	Th_17_ cells [87]
		Breast cancer [18]	Dendritic Cells [88]
		Hepatocellular carcinoma [18]	
		Tyroid cancer [86]	
		Ovarian cancer [86]	
		Cutaneous T cell lymphoma [89]	
		Laringeal cancer [90]	
CCR7	CCL19 CCL21	Breast cancer [3]	T cells [91]
		Gastric cancer [3,92]	Th_22_ T cells [57]
		Colorectal cancer [3]	T_reg_ cells [91]
		Lung cancer [3,93]	Dendritic Cells [91]
		Esophageal cancer [3,6]	B cells [91]
		Leukemia [3,94]	
CCR8	CCL1 CCL4 CCL17 CCL18	Breast cancer [95]	
CCR9	CCL25	Melanoma [96]	
		Prostate cancer [97]	
CCR10	CCL27 CCL28	Leukemia [98,99]	T_reg_ cells [100]
		Melanoma [101]	
CX3CR1	CX3CL1	Pancreatic cancer [102,103]	TAMs [104]
		Prostate cancer [105]	
		Breast cancer [106]	
ACKR3 (CXCR7)	CXCL11 CXCL12	Breast cancer [107]	Endothelial cells [108]
		Prostate cancer [109]	
ACKR5 (CCRL2)	CCL2 CCL5 CCL7 CCL8 CCL19	Colorectal cancer [39]	
**?**	CXCL14		Dendritic Cells [110]
