# Correction: Marcuzzi, E., et al. Chemokines and Chemokine Receptors: Orchestrating Tumor Metastasization. *Int. J. Mol. Sci.* 2019, *20*, 96

**DOI:** 10.3390/ijms20112651

**Published:** 2019-05-29

**Authors:** Elisabetta Marcuzzi, Roberta Angioni, Barbara Molon, Bianca Calì

**Affiliations:** 1 Department of Biomedical Sciences, University of Padova, 35131 Padova, Italy; elisabetta.marcuzzi@phd.unipd.it (E.M.); roberta.angioni@studenti.unipd.it (R.A.); barbara.molon@unipd.it (B.M.); 2 Fondazione Istituto di Ricerca Pediatrica Città della Speranza, 35127 Padova, Italy; 3 Institut Curie, 75005 Paris, France

Due to a typesetting error during layout, several references were incorrectly listed in [1]. In Table 1, citations to references 18, 24–28, 30–34, 36, 38–41, 47–51, 53–55, 63–69, 76–87, 89–101, 104–107, and 110 were incorrect. In the references list, references 47–214 were incorrectly numbered. The authors wish to make the following corrections to this paper [1]. The references list has been updated accordingly.

Reference 18 to “Song, J.K.; Park, M.H.; Choi, D.Y.; Yoo, H.S.; Han, S.B.; Yoon, D.Y.; Hong, J.T. Deficiency of C-C chemokine receptor 5 suppresses tumor development via inactivation of NF-kappaB and upregulation of IL-1Ra in melanoma model. *PLoS ONE* **2012**, *7*, e33747.” is incorrect. The correct reference is “19. O’Hayre, M.; Salanga, C.L.; Handel, T.M.; Allen, S.J. Chemokines and cancer: Migration, intracellular signaling and intercellular communication in the microenvironment. Biochem. J. 2008, 409, 635–649.”

Reference 24 to “Jung, S.J.; Kim, C.I.; Park, C.H.; Chang, H.S.; Kim, B.H.; Choi, M.S.; Jung, H.R., Correlation between Chemokine Receptor CXCR4 Expression and Prognostic Factors in Patients with Prostate Cancer. Korean J. Urol. 2011, 52, 607–611.” should be correctly numbered as 25.

Reference 25 to “Zhao, B.C.; Wang, Z.J.; Mao, W.Z.; Ma, H.C.; Han, J.G.; Zhao, B.; Xu, H.M. CXCR4/SDF-1 axis is involved in lymph node metastasis of gastric carcinoma. World J. Gastroenterol. 2011, 17, 2389–2396.” is incorrect. The correct reference is “26. Kaifi, J.T.; Yekebas, E.F.; Schurr, P.; Obonyo, D.; Wachowiak, R.; Busch, P.; Heinecke, A.; Pantel, K.; Izbicki, J.R., Tumor-cell homing to lymph nodes and bone marrow and CXCR4 expression in esophageal cancer. J. Natl. Cancer Inst. 2005, 97, 1840–1847.”

Reference 26 to “Kaifi, J.T.; Yekebas, E.F.; Schurr, P.; Obonyo, D.; Wachowiak, R.; Busch, P.; Heinecke, A.; Pantel, K.; Izbicki, J.R., Tumor-cell homing to lymph nodes and bone marrow and CXCR4 expression in esophageal cancer. J. Natl. Cancer Inst. 2005, 97, 1840–1847” is incorrect. The correct reference is “27. Kajiyama, H.; Shibata, K.; Terauchi, M.; Ino, K.; Nawa, A.; Kikkawa, F., Involvement of SDF-1alpha/CXCR4 axis in the enhanced peritoneal metastasis of epithelial ovarian carcinoma. Int. J. Cancer 2008, 122, 91–99.”

Reference 27 to “Kajiyama, H.; Shibata, K.; Terauchi, M.; Ino, K.; Nawa, A.; Kikkawa, F., Involvement of SDF-1alpha/CXCR4 axis in the enhanced peritoneal metastasis of epithelial ovarian carcinoma. Int. J. Cancer 2008, 122, 91–99.” is incorrect. The correct reference is “28. Liu, Q.; Li, A.; Tian, Y.; Wu, J.D.; Liu, Y.; Li, T.; Chen, Y.; Han, X.; Wu, K. The CXCL8-CXCR1/2 pathways in cancer. Cytokine Growth Factor Rev. 2016, 31, 61–71.”

Reference 28 to “Liu, Q.; Li, A.; Tian, Y.; Wu, J.D.; Liu, Y.; Li, T.; Chen, Y.; Han, X.; Wu, K. The CXCL8-CXCR1/2 pathways in cancer. Cytokine Growth Factor Rev. 2016, 31, 61–71.” is incorrect. The correct reference is “29. Du, L.; Han, X.G.; Tu, B.; Wang, M.Q.; Qiao, H.; Zhang, S.H.; Fan, Q.M.; Tang, T.T. CXCR1/Akt signaling activation induced by mesenchymal stem cell-derived IL-8 promotes osteosarcoma cell anoikis resistance and pulmonary metastasis. Cell Death Dis. 2018, 9, 714.”

Reference 33 to “Scheibenbogen, C.; Mohler, T.; Haefele, J.; Hunstein, W.; Keilholz, U. Serum interleukin-8 (IL-8) is elevated in patients with metastatic melanoma and correlates with tumour load. Melanoma Res. 1995, 5, 179–181.” is incorrect. The correct reference is “34. Doll, D.; Keller, L.; Maak, M.; Boulesteix, A.L.; Siewert, J.R.; Holzmann, B.; Janssen, K.P. Differential expression of the chemokines GRO-2, GRO-3, and interleukin-8 in colon cancer and their impact on metastatic disease and survival. Int. J. Colorectal. Dis. 2010, 25, 573–581.”

References 30–32 are incorrect. The correct ones are the following: “32. Nurnberg, W.; Tobias, D.; Otto, F.; Henz, B.M.; Schadendorf, D. Expression of interleukin-8 detected by in situ hybridization correlates with worse prognosis in primary cutaneous melanoma. J. Pathol. 1999, 189, 546–551.”; “33. Scheibenbogen, C.; Mohler, T.; Haefele, J.; Hunstein, W.; Keilholz, U. Serum interleukin-8 (IL-8) is elevated in patients with metastatic melanoma and correlates with tumour load. Melanoma Res. 1995, 5, 179–181.”; and “60. Balkwill, F.; Charles, K.A.; Mantovani, A. Smoldering and polarized inflammation in the initiation and promotion of malignant disease. Cancer Cell 2005, 7, 211–217.”

Reference 34 to “Doll, D.; Keller, L.; Maak, M.; Boulesteix, A.L.; Siewert, J.R.; Holzmann, B.; Janssen, K.P. Differential expression of the chemokines GRO-2, GRO-3, and interleukin-8 in colon cancer and their impact on metastatic disease and survival. Int. J. Colorectal. Dis. 2010, 25, 573–581.” is incorrect. The correct reference is “19. O’Hayre, M.; Salanga, C.L.; Handel, T.M.; Allen, S.J. Chemokines and cancer: Migration, intracellular signalling and intercellular communication in the microenvironment. Biochem. J. 2008, 409, 635–649”. 

Reference 36 to “Kawada, K.; Sonoshita, M.; Sakashita, H.; Takabayashi, A.; Yamaoka, Y.; Manabe, T.; Inaba, K.; Minato, N.; Oshima, M.; Taketo, M.M. Pivotal role of CXCR3 in melanoma cell metastasis to lymph nodes. Cancer Res. 2004, 64, 4010–4017.” is incorrect. The correct reference is “37. Ma, X.; Norsworthy, K.; Kundu, N.; Rodgers, W.H.; Gimotty, P.A.; Goloubeva, O.; Lipsky, M.; Li, Y.; Holt, D.; Fulton, A. CXCR3 expression is associated with poor survival in breast cancer and promotes metastasis in a murine model. Mol. Cancer Ther. 2009, 8, 490–498.”

Reference 38 to “Muller, G.; Hopken, U.E.; Lipp, M., The impact of CCR7 and CXCR5 on lymphoid organ development and systemic immunity. Immunol. Rev. 2003, 195, 117–135.” is incorrect. The correct reference is “39. Laverdiere, C.; Hoang, B.H.; Yang, R.; Sowers, R.; Qin, J.; Meyers, P.A.; Huvos, A.G.; Healey, J.H.; Gorlick, R. Messenger RNA expression levels of CXCR4 correlate with metastatic behavior and outcome in patients with osteosarcoma. Clin. Cancer Res. 2005, 11, 2561–2567.”

Reference 39 to “Laverdiere, C.; Hoang, B.H.; Yang, R.; Sowers, R.; Qin, J.; Meyers, P.A.; Huvos, A.G.; Healey, J.H.; Gorlick, R. Messenger RNA expression levels of CXCR4 correlate with metastatic behavior and outcome in patients with osteosarcoma. Clin. Cancer Res. 2005, 11, 2561–2567.” is incorrect. The correct reference is “40. Akram, I.G.; Georges, R.; Hielscher, T.; Adwan, H.; Berger, M.R. The chemokines CCR1 and CCRL2 have a role in colorectal cancer liver metastasis. Tumour. Biol. 2016, 37, 2461–2471.”

Reference 40 to “Akram, I.G.; Georges, R.; Hielscher, T.; Adwan, H.; Berger, M.R. The chemokines CCR1 and CCRL2 have a role in colorectal cancer liver metastasis. Tumour. Biol. 2016, 37, 2461–2471.” is incorrect. The correct reference is “41. Hirai, H.; Fujishita, T.; Kurimoto, K.; Miyachi, H.; Kitano, S.; Inamoto, S.; Itatani, Y.; Saitou, M.; Maekawa, T.; Taketo, M.M. CCR1-mediated accumulation of myeloid cells in the liver microenvironment promoting mouse colon cancer metastasis. Clin. Exp. Metastasis 2014, 31, 977–989.”

Reference 41 to “Hirai, H.; Fujishita, T.; Kurimoto, K.; Miyachi, H.; Kitano, S.; Inamoto, S.; Itatani, Y.; Saitou, M.; Maekawa, T.; Taketo, M.M. CCR1-mediated accumulation of myeloid cells in the liver microenvironment promoting mouse colon cancer metastasis. Clin. Exp. Metastasis 2014, 31, 977–989.” is incorrect. The correct reference is “42. Shin, S.Y.; Lee, D.H.; Lee, J.; Choi, C.; Kim, J.Y.; Nam, J.S.; Lim, Y.; Lee, Y.H. C-C motif chemokine receptor 1 (CCR1) is a target of the EGF-AKT-mTOR-STAT3 signaling axis in breast cancer cells. Oncotarget 2017, 8, 94591–94605.”

Reference 47 to “Molon, B.; Ugel, S.; Del Pozzo, F.; Soldani, C.; Zilio, S.; Avella, D.; De Palma, A.; Mauri, P.; Monegal, A.; Rescigno, M.; et al. Chemokine nitration prevents intratumoral infiltration of antigen-specific T cells. J. Exp. Med. 2011, 208, 1949–1962.” is incorrect. The correct reference is “Mantovani, A.; Sica, A.; Sozzani, S.; Allavena, P.; Vecchi, A.; Locati, M. The chemokine system in diverse forms of macrophage activation and polarization. Trends Immunol. 2004, 25, 677–686.”

Reference 48 to “Grivennikov, S.I.; Greten, F.R.; Karin, M., Immunity, inflammation, and cancer. Cell 2010, 140, (6), 883–899.” is incorrect. The correct reference is “Obermajer, N.; Muthuswamy, R.; Odunsi, K.; Edwards, R.P.; Kalinski, P. PGE(2)-induced CXCL12 production and CXCR4 expression controls the accumulation of human MDSCs in ovarian cancer environment. *Cancer Res.* **2011**, *71*, 7463–7470”.

Reference 49 to “Kryczek, I.; Wei, S.; Gong, W.; Shu, X.; Szeliga, W.; Vatan, L.; Chen, L.; Wang, G.; Zou, W. Cutting edge: IFN-gamma enables APC to promote memory Th17 and abate Th1 cell development. J. Immunol. 2008, 181, 5842–5846.” is incorrect. The correct reference is “Jablonska, J.; Lang, S.; Sionov, R.V.; Granot, Z. The regulation of pre-metastatic niche formation by neutrophils. Oncotarget 2017, 8, 112132–112144.”

Reference 50 to “Liekens, S.; Schols, D.; Hatse, S. CXCL12-CXCR4 axis in angiogenesis, metastasis and stem cell mobilization. Curr. Pharm. Des. 2010, 16, 3903–3920.” is incorrect. The correct reference is “Kanda, S.; Mochizuki, Y.; Kanetake, H. Stromal cell-derived factor-1alpha induces tube-like structure formation of endothelial cells through phosphoinositide 3-kinase. J. Biol. Chem. 2003, 278, 257–262.”

Reference 51 to “Kanda, S.; Mochizuki, Y.; Kanetake, H. Stromal cell-derived factor-1alpha induces tube-like structure formation of endothelial cells through phosphoinositide 3-kinase. J. Biol. Chem. 2003, 278, 257–262” is incorrect. The correct reference is “Liekens, S.; Schols, D.; Hatse, S. CXCL12-CXCR4 axis in angiogenesis, metastasis and stem cell mobilization. Curr. Pharm. Des. 2010, 16, 3903–3920.”

Reference 53 to “Kitamura, T.; Qian, B.Z.; Soong, D.; Cassetta, L.; Noy, R.; Sugano, G.; Kato, Y.; Li, J.; Pollard, J.W. CCL2-induced chemokine cascade promotes breast cancer metastasis by enhancing retention of metastasis-associated macrophages. J. Exp. Med. 2015, 212, 1043–1059.” is incorrect. The correct reference is “Granot, Z.; Henke, E.; Comen, E.A.; King, T.A.; Norton, L.; Benezra, R. Tumor entrained neutrophils inhibit seeding in the premetastatic lung. Cancer Cell 2011, 20, 300–314.”

Reference 54 to “Bell, D.; Chomarat, P.; Broyles, D.; Netto, G.; Harb, G.M.; Lebecque, S.; Valladeau, J.; Davoust, J.; Palucka, K.A.; Banchereau, J. In breast carcinoma tissue, immature dendritic cells reside within the tumor, whereas mature dendritic cells are located in peritumoral areas. J. Exp. Med. 1999, 190, 1417–1426.” is incorrect. The correct reference is “Condamine, T.; Ramachandran, I.; Youn, J.I.; Gabrilovich, D.I. Regulation of tumor metastasis by myeloid-derived suppressor cells. Annu. Rev. Med. 2015, 66, 97–110.”

Reference 55 to “Curiel, T.J.; Coukos, G.; Zou, L.; Alvarez, X.; Cheng, P.; Mottram, P.; Evdemon-Hogan, M.; Conejo-Garcia, J.R.; Zhang, L.; Burow, M.; et al. Specific recruitment of regulatory T cells in ovarian carcinoma fosters immune privilege and predicts reduced survival. Nat. Med. 2004, 10, 942–949.” is incorrect. The correct reference is “Wang, Z.; Liu, H.; Shen, Z.; Wang, X.; Zhang, H.; Qin, J.; Xu, J.; Sun, Y.; Qin, X. The prognostic value of CXC-chemokine receptor 2 (CXCR2) in gastric cancer patients. BMC Cancer 2015, 15, 766.”

Reference to “Pinedo, H.M.; Verheul, H.M.; D’Amato, R J.; Folkman, J. Involvement of platelets in tumour angiogenesis? Lancet 1998, 352, (9142), 1775-7.” is incorrect. The correct reference is “Pages, F.; Berger, A.; Camus, M.; Sanchez-Cabo, F.; Costes, A.; Molidor, R.; Mlecnik, B.; Kirilovsky, A.; Nilsson, M.; Damotte, D.; Meatchi, T.; Bruneval, P.; Cugnenc, P. H.; Trajanoski, Z.; Fridman, W. H.; Galon, J., Effector memory T cells, early metastasis, and survival in colorectal cancer. N. Engl. J. Med. 2005, 353, (25), 2654-66.”

Reference 64 to “Leblanc, R.; Peyruchaud, O., Metastasis: new functional implications of platelets and megakaryocytes. Blood 2016, 128, (1), 24-31.” is incorrect. The correct reference is “Zhang, L.; Conejo-Garcia, J.R.; Katsaros, D.; Gimotty, P.A.; Massobrio, M.; Regnani, G.; Makrigiannakis, A.; Gray, H.; Schlienger, K.; Liebman, M. N.; Rubin, S.C.; Coukos, G., Intratumoral T cells, recurrence, and survival in epithelial ovarian cancer. N. Engl. J. Med. 2003, 348, (3), 203-13.”

Reference 65 to “Cools-Lartigue, J.; Spicer, J.; McDonald, B.; Gowing, S.; Chow, S.; Giannias, B.; Bourdeau, F.; Kubes, P.; Ferri, L. Neutrophil extracellular traps sequester circulating tumor cells and promote metastasis. J. Clin. Investig. 2013.” is incorrect. The correct reference is “Soria, G.; Ben-Baruch, A., The inflammatory chemokines CCL2 and CCL5 in breast cancer. Cancer Lett. 2008, 267, 271–285.”

Reference 67 to “Albrengues, J.; Shields, M.A.; Ng, D.; Park, C.G.; Ambrico, A.; Poindexter, M.E.; Upadhyay, P.; Uyeminami, D.L.; Pommier, A.; Kuttner, V.; et al. Neutrophil extracellular traps produced during inflammation awaken dormant cancer cells in mice. Science 2018, 361.” is incorrect. The correct reference is “Condamine, T.; Ramachandran, I.; Youn, J.I.; Gabrilovich, D.I. Regulation of tumor metastasis by myeloid-derived suppressor cells. Annu. Rev. Med. 2015, 66, 97–110.”

Reference 68 to “Spiegel, A.; Brooks, M.W.; Houshyar, S.; Reinhardt, F.; Ardolino, M.; Fessler, E.; Chen, M.B.; Krall, J.A.; DeCock, J.; Zervantonakis, I.K.; et al. Neutrophils Suppress Intraluminal NK Cell-Mediated Tumor Cell Clearance and Enhance Extravasation of Disseminated Carcinoma Cells. Cancer Discov. 2016, 6, 630–649.” is incorrect. The correct reference is “Molon, B.; Ugel, S.; Del Pozzo, F.; Soldani, C.; Zilio, S.; Avella, D.; De Palma, A.; Mauri, P.; Monegal, A.; Rescigno, M.; et al. Chemokine nitration prevents intratumoral infiltration of antigen-specific T cells. J. Exp. Med. 2011, 208, 1949–1962.”

Reference 69 to “McDonald, B.; Spicer, J.; Giannais, B.; Fallavollita, L.; Brodt, P.; Ferri, L.E. Systemic inflammation increases cancer cell adhesion to hepatic sinusoids by neutrophil mediated mechanisms. Int. J. Cancer 2009, 125, 1298–1305.” is incorrect. The correct reference is “Qian, B.Z.; Li, J.; Zhang, H.; Kitamura, T.; Zhang, J.; Campion, L.R.; Kaiser, E.A.; Snyder, L.A.; Pollard, J.W. CCL2 recruits inflammatory monocytes to facilitate breast-tumour metastasis. Nature 2011, 475, 222–225.”

Reference 76 to “Huang, B.; Lei, Z.; Zhao, J.; Gong, W.; Liu, J.; Chen, Z.; Liu, Y.; Li, D.; Yuan, Y.; Zhang, G.M.; et al. CCL2/CCR2 pathway mediates recruitment of myeloid suppressor cells to cancers. Cancer Lett. 2007, 252, 86–92.“ is incorrect. The correct reference is “Kitamura, T.; Qian, B.Z.; Soong, D.; Cassetta, L.; Noy, R.; Sugano, G.; Kato, Y.; Li, J.; Pollard, J.W. CCL2-induced chemokine cascade promotes breast cancer metastasis by enhancing retention of metastasis-associated macrophages. J. Exp. Med. 2015, 212, 1043–1059.”

Reference 77 to “Kleinhans, M.; Tun-Kyi, A.; Gilliet, M.; Kadin, M.E.; Dummer, R.; Burg, G.; Nestle, F.O. Functional expression of the eotaxin receptor CCR3 in CD30+ cutaneous T-cell lymphoma. Blood 2003, 101, 1487–1493.” is incorrect. The correct reference is “Slettenaar, V.I.; Wilson, J.L. The chemokine network: A target in cancer biology? Adv. Drug. Deliv. Rev. 2006, 58, 962–974.”

Reference 78 to “Stoll, G.; Pol, J.; Soumelis, V.; Zitvogel, L.; Kroemer, G. Impact of chemotactic factors and receptors on the cancer immune infiltrate: A bioinformatics study revealing homogeneity and heterogeneity among patient cohorts. Oncoimmunology 2018, 7, e1484980.” is incorrect. The correct reference is “Curiel, T.J.; Coukos, G.; Zou, L.; Alvarez, X.; Cheng, P.; Mottram, P.; Evdemon-Hogan, M.; Conejo-Garcia, J.R.; Zhang, L.; Burow, M.; et al. Specific recruitment of regulatory T cells in ovarian carcinoma fosters immune privilege and predicts reduced survival. Nat. Med. 2004, 10, 942–949.”

Reference 79 to “Ishida, T.; Ueda, R., CCR4 as a novel molecular target for immunotherapy of cancer. Cancer Sci. 2006, 97, 1139–1146.“ is incorrect. The correct reference is “Li, J.Y.; Ou, Z.L.; Yu, S.J.; Gu, X.L.; Yang, C.; Chen, A.X.; Di, G.H.; Shen, Z.Z.; Shao, Z.M. The chemokine receptor CCR4 promotes tumor growth and lung metastasis in breast cancer. Breast Cancer Res. Treat 2012, 131, 837–848.”

Reference 80 to “Slettenaar, V.I.; Wilson, J.L. The chemokine network: A target in cancer biology? Adv. Drug. Deliv. Rev. 2006, 58, 962–974.” is incorrect. The correct reference is “Klein, A.; Sagi-Assif, O.; Meshel, T.; Telerman, A.; Izraely, S.; Ben-Menachem, S.; Bayry, J.; Marzese, D.M.; Ohe, S.; Hoon, D.S.B.; et al. CCR4 is a determinant of melanoma brain metastasis. Oncotarget 2017, 8, 31079–31091.”

Reference 81 to “Li, J.Y.; Ou, Z.L.; Yu, S.J.; Gu, X.L.; Yang, C.; Chen, A.X.; Di, G.H.; Shen, Z.Z.; Shao, Z.M. The chemokine receptor CCR4 promotes tumor growth and lung metastasis in breast cancer. Breast Cancer Res. Treat 2012, 131, 837–848.“ is incorrect. The correct reference is “Cheng, X.; Wu, H.; Jin, Z.J.; Ma, D.; Yuen, S.; Jing, X.Q.; Shi, M.M.; Shen, B.Y.; Peng, C.H.; Zhao, R.; et al. Up-regulation of chemokine receptor CCR4 is associated with Human Hepatocellular Carcinoma malignant behavior. Sci. Rep. 2017, 7, 12362.”

Reference 82 to “Kryczek, I.; Lin, Y.; Nagarsheth, N.; Peng, D.; Zhao, L.; Zhao, E.; Vatan, L.; Szeliga, W.; Dou, Y.; Owens, S.; et al. IL-22(+)CD4(+) T cells promote colorectal cancer stemness via STAT3 transcription factor activation and induction of the methyltransferase DOT1L. Immunity. 2014, 40, 772–784.” is incorrect. The correct reference is “Soria, G.; Ben-Baruch, A., The inflammatory chemokines CCL2 and CCL5 in breast cancer. Cancer Lett. 2008, 267, 271–285.”

Reference 83 to “Kryczek, I.; Banerjee, M.; Cheng, P.; Vatan, L.; Szeliga, W.; Wei, S.; Huang, E.; Finlayson, E.; Simeone, D.; Welling, T.H.; et al. Phenotype, distribution, generation, and functional and clinical relevance of Th17 cells in the human tumor environments. Blood 2009, 114, 1141–1149.” is incorrect. The correct reference is “Stoll, G.; Pol, J.; Soumelis, V.; Zitvogel, L.; Kroemer, G., Impact of chemotactic factors and receptors on the cancer immune infiltrate: a bioinformatics study revealing homogeneity and heterogeneity among patient cohorts. Oncoimmunology 2018, 7, (10), e1484980.”

Reference 84 to “Klein, A.; Sagi-Assif, O.; Meshel, T.; Telerman, A.; Izraely, S.; Ben-Menachem, S.; Bayry, J.; Marzese, D.M.; Ohe, S.; Hoon, D.S.B.; et al. CCR4 is a determinant of melanoma brain metastasis. Oncotarget 2017, 8, 31079–31091.“ is incorrect. The correct reference is “von Luettichau, I.; Nathrath, M.; Burdach, S.; Huss, R.; Segerer, S.; Nelson, P.J. Mononuclear infiltrates in osteosarcoma and chemokine receptor expression. Clin. Cancer Res. 2006, 12, 5253–5254.”

Reference 85 to “Cheng, X.; Wu, H.; Jin, Z.J.; Ma, D.; Yuen, S.; Jing, X.Q.; Shi, M.M.; Shen, B.Y.; Peng, C.H.; Zhao, R.; et al. Up-regulation of chemokine receptor CCR4 is associated with Human Hepatocellular Carcinoma malignant behavior. Sci. Rep. 2017, 7, 12362.” is incorrect. The correct reference is “Dellacasagrande, J.; Schreurs, O.J.; Hofgaard, P.O.; Omholt, H.; Steinsvoll, S.; Schenck, K.; Bogen, B.; Dembic, Z. Liver metastasis of cancer facilitated by chemokine receptor CCR6. Scand. J. Immunol. 2003, 57, 534–544.”

Reference 86 to “von Luettichau, I.; Nathrath, M.; Burdach, S.; Huss, R.; Segerer, S.; Nelson, P.J. Mononuclear infiltrates in osteosarcoma and chemokine receptor expression. Clin. Cancer Res. 2006, 12, 5253–5254.” is incorrect. The correct reference is “Liu, J.; Ke, F.; Xu, Z.; Liu, Z.; Zhang, L.; Yan, S.; Wang, Z.; Wang, H.; Wang, H. CCR6 is a prognostic marker for overall survival in patients with colorectal cancer, and its overexpression enhances metastasis in vivo. PLoS ONE 2014, 9, e101137”.

Reference 87 to “Ward, Y.; Lake, R.; Faraji, F.; Sperger, J.; Martin, P.; Gilliard, C.; Ku, K.P.; Rodems, T.; Niles, D.; Tillman, H.; et al. Platelets Promote Metastasis via Binding Tumor CD97 Leading to Bidirectional Signaling that Coordinates Transendothelial Migration. Cell Rep. 2018, 23, 808–822.” is incorrect. The correct reference is “Kryczek, I.; Banerjee, M.; Cheng, P.; Vatan, L.; Szeliga, W.; Wei, S.; Huang, E.; Finlayson, E.; Simeone, D.; Welling, T.H.; et al. Phenotype, distribution, generation, and functional and clinical relevance of Th17 cells in the human tumor environments. Blood 2009, 114, 1141–1149.”

Reference 89 to “Liu, J.; Ke, F.; Xu, Z.; Liu, Z.; Zhang, L.; Yan, S.; Wang, Z.; Wang, H.; Wang, H. CCR6 is a prognostic marker for overall survival in patients with colorectal cancer, and its overexpression enhances metastasis in vivo. PLoS ONE 2014, 9, e101137.“ is incorrect. The correct reference is “Ikeda, S.; Kitadate, A.; Ito, M.; Abe, F.; Nara, M.; Watanabe, A.; Takahashi, N.; Miyagaki, T.; Sugaya, M.; Tagawa, H. Disruption of CCL20-CCR6 interaction inhibits metastasis of advanced cutaneous T-cell lymphoma. Oncotarget 2016, 7, 13563–13574.”

Reference 90 to “Dellacasagrande, J.; Schreurs, O.J.; Hofgaard, P.O.; Omholt, H.; Steinsvoll, S.; Schenck, K.; Bogen, B.; Dembic, Z. Liver metastasis of cancer facilitated by chemokine receptor CCR6. Scand. J. Immunol. 2003, 57, 534–544.” is incorrect. The correct reference is “Lu, E.; Su, J.; Zhou, Y.; Zhang, C.; Wang, Y. CCL20/CCR6 promotes cell proliferation and metastasis in laryngeal cancer by activating p38 pathway. Biomed. Pharmacother. 2017, 85, 486–492.”

Reference 91 to “De Sanctis, F.; Bronte, V.; Ugel, S. Tumor-Induced Myeloid-Derived Suppressor Cells. Microbiol. Spectr. 2016, 4.” is incorrect. The correct reference is “DeGregori, J., Connecting Cancer to Its Causes Requires Incorporation of Effects on Tissue Microenvironments. Cancer Res. 2017, 77, (22), 6065-6068.”

Reference 92 to “Lu, E.; Su, J.; Zhou, Y.; Zhang, C.; Wang, Y. CCL20/CCR6 promotes cell proliferation and metastasis in laryngeal cancer by activating p38 pathway. Biomed Pharmacother. 2017, 85, 486–492.” is incorrect. The correct reference is “Mashino, K.; Sadanaga, N.; Yamaguchi, H.; Tanaka, F.; Ohta, M.; Shibuta, K.; Inoue, H.; Mori, M. Expression of chemokine receptor CCR7 is associated with lymph node metastasis of gastric carcinoma. Cancer Res. 2002, 62, 2937–2941.”

Reference 93 to “Boyle, S.T.; Faulkner, J.W.; McColl, S.R.; Kochetkova, M. The chemokine receptor CCR6 facilitates the onset of mammary neoplasia in the MMTV-PyMT mouse model via recruitment of tumor-promoting macrophages. Mol. Cancer 2015, 14, 115.” is incorrect. The correct reference is “Takanami, I., Overexpression of CCR7 mRNA in nonsmall cell lung cancer: Correlation with lymph node metastasis. Int. J. Cancer 2003, 105, 186–189.”

Reference 94 to “Takanami, I., Overexpression of CCR7 mRNA in nonsmall cell lung cancer: Correlation with lymph node metastasis. Int. J. Cancer 2003, 105, 186–189.” is incorrect. The correct reference is “Till, K.J.; Lin, K.; Zuzel, M.; Cawley, J.C., The chemokine receptor CCR7 and alpha4 integrin are important for migration of chronic lymphocytic leukemia cells into lymph nodes. Blood 2002, 99, 2977–2984.”

Reference 95 to “Mantovani, A.; Sozzani, S.; Locati, M.; Allavena, P.; Sica, A. Macrophage polarization: Tumor-associated macrophages as a paradigm for polarized M2 mononuclear phagocytes. Trends Immunol. 2002, 23, 549–555.” is incorrect. The correct reference is “Das, S.; Sarrou, E.; Podgrabinska, S.; Cassella, M.; Mungamuri, S.K.; Feirt, N.; Gordon, R.; Nagi, C.S.; Wang, Y.; Entenberg, D.; et al. Tumor cell entry into the lymph node is controlled by CCL1 chemokine expressed by lymph node lymphatic sinuses. J. Exp. Med. 2013, 210, 1509–1528.”

Reference 96 to “Ding, Y.; Shimada, Y.; Maeda, M.; Kawabe, A.; Kaganoi, J.; Komoto, I.; Hashimoto, Y.; Miyake, M.; Hashida, H.; Imamura, M. Association of CC chemokine receptor 7 with lymph node metastasis of esophageal squamous cell carcinoma. Clin. Cancer Res. 2003, 9, 3406–3412.” is incorrect. The correct reference is “Amersi, F.F.; Terando, A.M.; Goto, Y.; Scolyer, R.A.; Thompson, J.F.; Tran, A.N.; Faries, M.B.; Morton, D.L.; Hoon, D.S., Activation of CCR9/CCL25 in cutaneous melanoma mediates preferential metastasis to the small intestine. Clin. Cancer Res. 2008, 14, 638–645.”

Reference 97 to “Amersi, F.F.; Terando, A.M.; Goto, Y.; Scolyer, R.A.; Thompson, J.F.; Tran, A.N.; Faries, M.B.; Morton, D.L.; Hoon, D.S., Activation of CCR9/CCL25 in cutaneous melanoma mediates preferential metastasis to the small intestine. Clin. Cancer Res. 2008, 14, 638–645.” is incorrect. The correct reference is “Singh, R.; Stockard, C.R.; Grizzle, W.E.; Lillard, J.W., Jr.; Singh, S., Expression and histopathological correlation of CCR9 and CCL25 in ovarian cancer. Int. J. Oncol. 2011, 39, 373–381.”

Reference 98 to “Singh, R.; Stockard, C.R.; Grizzle, W.E.; Lillard, J.W., Jr.; Singh, S., Expression and histopathological correlation of CCR9 and CCL25 in ovarian cancer. Int. J. Oncol. 2011, 39, 373–381.” is incorrect. The correct reference is “Harasawa, H.; Yamada, Y.; Hieshima, K.; Jin, Z.; Nakayama, T.; Yoshie, O.; Shimizu, K.; Hasegawa, H.; Hayashi, T.; Imaizumi, Y.; et al. Survey of chemokine receptor expression reveals frequent co-expression of skin-homing CCR4 and CCR10 in adult T-cell leukemia/lymphoma. Leuk. Lymphoma 2006, 47, 2163–2173.”

Reference 99 to “Lazennec, G.; Richmond, A., Chemokines and chemokine receptors: New insights into cancer-related inflammation. Trends Mol. Med. 2010, 16, 133–144.” is incorrect. The correct reference is “Notohamiprodjo, M.; Segerer, S.; Huss, R.; Hildebrandt, B.; Soler, D.; Djafarzadeh, R.; Buck, W.; Nelson, P.J.; von Luettichau, I., CCR10 is expressed in cutaneous T-cell lymphoma. Int. J. Cancer 2005, 115, 641–647.”

Reference 100 to “Ohl, L.; Bernhardt, G.; Pabst, O.; Forster, R., Chemokines as organizers of primary and secondary lymphoid organs. Semin. Immunol. 2003, 15, 249–255.” is incorrect. The correct reference is “Facciabene, A.; Peng, X.; Hagemann, I.S.; Balint, K.; Barchetti, A.; Wang, L.P.; Gimotty, P.A.; Gilks, C.B.; Lal, P.; Zhang, L.; et al. Tumour hypoxia promotes tolerance and angiogenesis via CCL28 and T(reg) cells. Nature 2011, 475, 226–230.”

Reference 101 to “Till, K.J.; Lin, K.; Zuzel, M.; Cawley, J.C., The chemokine receptor CCR7 and alpha4 integrin are important for migration of chronic lymphocytic leukemia cells into lymph nodes. Blood 2002, 99, 2977–2984.” is incorrect. The correct reference is “Lazennec, G.; Richmond, A., Chemokines and chemokine receptors: New insights into cancer-related inflammation. Trends Mol. Med. 2010, 16, 133–144.”

Reference 104 to “Qian, B.Z.; Li, J.; Zhang, H.; Kitamura, T.; Zhang, J.; Campion, L.R.; Kaiser, E.A.; Snyder, L.A.; Pollard, J.W. CCL2 recruits inflammatory monocytes to facilitate breast-tumour metastasis. Nature 2011, 475, 222–225.” is incorrect. The correct reference is “Zheng, J.; Yang, M.; Shao, J.; Miao, Y.; Han, J.; Du, J. Chemokine receptor CX3CR1 contributes to macrophage survival in tumor metastasis. Mol. Cancer 2013, 12, 141.”

Reference 105 to “Celesti, G.; Di Caro, G.; Bianchi, P.; Grizzi, F.; Marchesi, F.; Basso, G.; Rahal, D.; Delconte, G.; Catalano, M.; Cappello, P.; et al. Early expression of the fractalkine receptor CX3CR1 in pancreatic carcinogenesis. Br. J. Cancer 2013, 109, 2424–2433.” is incorrect. The correct reference is “Shulby, S.A.; Dolloff, N.G.; Stearns, M.E.; Meucci, O.; Fatatis, A. CX3CR1-fractalkine expression regulates cellular mechanisms involved in adhesion, migration, and survival of human prostate cancer cells. Cancer Res. 2004, 64, 4693–4698.”

Reference 106 to “Marchesi, F.; Piemonti, L.; Fedele, G.; Destro, A.; Roncalli, M.; Albarello, L.; Doglioni, C.; Anselmo, A.; Doni, A.; Bianchi, P.; et al. The chemokine receptor CX3CR1 is involved in the neural tropism and malignant behavior of pancreatic ductal adenocarcinoma. Cancer Res. 2008, 68, 9060–9069.” is incorrect. The correct reference is “Andre, F.; Cabioglu, N.; Assi, H.; Sabourin, J.C.; Delaloge, S.; Sahin, A.; Broglio, K.; Spano, J.P.; Combadiere, C.; Bucana, C.; et al. Expression of chemokine receptors predicts the site of metastatic relapse in patients with axillary node positive primary breast cancer. Ann. Oncol. 2006, 17, 945–951.”

Reference 107 to “Hansell, C.A.H.; Fraser, A.R.; Hayes, A.J.; Pingen, M.; Burt, C.L.; Lee, K.M.; Medina-Ruiz, L.; Brownlie, D.; Macleod, M.K.L.; Burgoyne, P.; et al. The Atypical Chemokine Receptor Ackr2 Constrains NK Cell Migratory Activity and Promotes Metastasis. J. Immunol. 2018, 201, 2510–2519.” is incorrect. The correct reference is “Zabel, B.A.; Wang, Y.; Lewen, S.; Berahovich, R.D.; Penfold, M.E.; Zhang, P.; Powers, J.; Summers, B.C.; Miao, Z.; Zhao, B.; et al. Elucidation of CXCR7-mediated signaling events and inhibition of CXCR4-mediated tumor cell transendothelial migration by CXCR7 ligands. J. Immunol. 2009, 183, 3204–3211.”

Reference 110 to “Hawila, E.; Razon, H.; Wildbaum, G.; Blattner, C.; Sapir, Y.; Shaked, Y.; Umansky, V.; Karin, N. CCR5 Directs the Mobilization of CD11b(+)Gr1(+)Ly6C(low) Polymorphonuclear Myeloid Cells from the Bone Marrow to the Blood to Support Tumor Development. Cell Rep. 2017, 21, 2212–2222.“ is incorrect. The correct reference is “Shurin, G.V.; Ferris, R.L.; Tourkova, I.L.; Perez, L.; Lokshin, A.; Balkir, L.; Collins, B.; Chatta, G.S.; Shurin, M.R., Loss of new chemokine CXCL14 in tumor tissue is associated with low infiltration by dendritic cells (DC), while restoration of human CXCL14 expression in tumor cells causes attraction of DC both in vitro and in vivo. J. Immunol. 2005, 174, 5490–5498.”

The manuscript will be updated and the original will remain online on the article webpage. The authors apologize for any inconvenience caused to the readers.
